# Case Report: Retroperitoneal bronchogenic cyst mimicking adrenal mass: Clinical implications and comprehensive literature analysis

**DOI:** 10.3389/fonc.2025.1679794

**Published:** 2025-10-01

**Authors:** Shengxiang Xiang, Jiange Hao, Xiaowen Zong, Shuang Wen, Jingxuan Zhang, Chunyu Chen, Jiayi Hao, Zhuwei Song, Heyao Tong, Liang Wang, Bo Fan, Zhiyu Liu

**Affiliations:** ^1^ Department of Urology, Second Affiliated Hospital of Dalian Medical University, Dalian, Liaoning, China; ^2^ Liaoning Provincial Key Laboratory of Urological Digital Precision Diagnosis and Treatment, Dalian, Liaoning, China; ^3^ Liaoning Engineering Research Center of Integrated Precision Diagnosis and Treatment Technology for Urological Cancer, Dalian, Liaoning, China; ^4^ Dalian Key Laboratory of Prostate Cancer Research, Dalian, Liaoning, China; ^5^ First Clinical College, Dalian Medical University, Dalian, Liaoning, China; ^6^ Department of Pathology, Dalian Friendship Hospital, Dalian, Liaoning, China; ^7^ College of Pharmacy, Zhongshan College of Dalian Medical University, Dalian, Liaoning, China

**Keywords:** bronchogenic cyst, retroperitoneal, adrenal mass, case report, robot-assisted laparoscopy surgery

## Abstract

**Background:**

Bronchogenic cysts are benign congenital anomalies originating from embryonic foregut development. Although typically located in the mediastinum or lung parenchyma, retroperitoneal bronchogenic cysts are exceedingly rare, with only a limited number of cases reported in the literature. We present a case of a bronchogenic cyst situated in the left retroperitoneal space, along with a concise review of previously documented cases, summarizing their common epidemiological, clinicopathological, and prognostic features.

**Case summary:**

A 48-year-old female was admitted with unexplained left lumbar pain. Computed tomography (CT) revealed a slightly hyperdense cystic mass measuring 43×37×36 mm in the left adrenal region. The lesion was successfully resected using robot-assisted laparoscopic surgery. Histopathological examination confirmed the diagnosis of a bronchogenic cyst. The patient remained hemodynamically stable and recovered well postoperatively. At the 6-month follow-up, she remained asymptomatic with no evidence of recurrence.

**Conclusion:**

Given the rarity of retroperitoneal bronchogenic cysts, preoperative imaging is often insufficient to definitively differentiate them from other retroperitoneal neoplasms. Consequently, definitive diagnosis usually depends on postoperative pathological evaluation. This case highlights the diagnostic challenge posed by this rare entity and underscores the importance of pathological examination for definitive diagnosis.

## Introduction

Bronchogenic cysts are relatively rare congenital malformations that typically originate from abnormal separation or migration of the trachea and bronchial buds during embryonic foregut development. These cysts are characteristically lined by respiratory epithelium (1). While commonly found in the mediastinum, retroperitoneal locations are exceedingly rare, first reported by Miller et al. in 1953 (2). Due to their generally asymptomatic nature, these cysts are often discovered incidentally on cross-sectional imaging such as CT or MRI. However, a small subset of patients may present with symptoms like pain or fever secondary to infection or mass effect (3, 4). Preoperative differentiating these cysts from other retroperitoneal lesions can be challenging. Surgical resection remains the preferred treatment, and definitive diagnosis is primarily established through histopathological analysis of the resected specimen (5). Herein, we present a case of a retroperitoneal bronchogenic cyst in a woman who initially presented with left lumbar pain and successfully underwent robot-assisted laparoscopic resection. Furthermore, we conduct a literature review to comprehensively summarize the clinical features of this rare disease, including its epidemiology, clinical manifestations, imaging characteristics, diagnostic approach, and treatment.

## Case presentation

A 48-year-old woman presented to her local hospital with a 3-month history of left lumbar pain and progressive weakness. During this evaluation, an unenhanced abdominal computed tomography (CT) scan incidentally identified a mass in the left adrenal region. To further characterize the retroperitoneal lesion, contrast-enhanced magnetic resonance imaging (MRI) scan of the adrenal region was performed. The MRI revealed an irregular mass measuring 43×31×42 mm, which exhibited slightly short T1 and long T2 signal characteristics and showed no contrast enhancement. Subsequently, the patient was referred to our institution for further diagnosis and treatment.

Adrenal mass needs to be differentiated from a variety of diseases, like primary aldosteronism, Cushing ‘s syndrome, pheochromocytoma, nonfunctional adrenal adenoma or adrenal tuberculosis. To exclude these possibilities, we conducted a detailed physical examination and specialized examinations on the patient. The patient denied other typical symptoms or signs such as central obesity, sudden dizziness, palpitations, sweating, nausea, vomiting, or fever. She reported adequate sleep, reduced appetite, normal voiding, and a weight loss of 2 kg over the previous two weeks. She denied any history of trauma, surgery, blood transfusion, or significant medical conditions including hypertension, diabetes, heart disease, or tuberculosis. There was no family history of malignancy or psychiatric illness. On physical examination, the abdomen was flat and soft, without tenderness or rebound. Percussion over the liver and kidney regions was non-tender. Overall, the physical examination findings were unremarkable.

**Figure 1:**
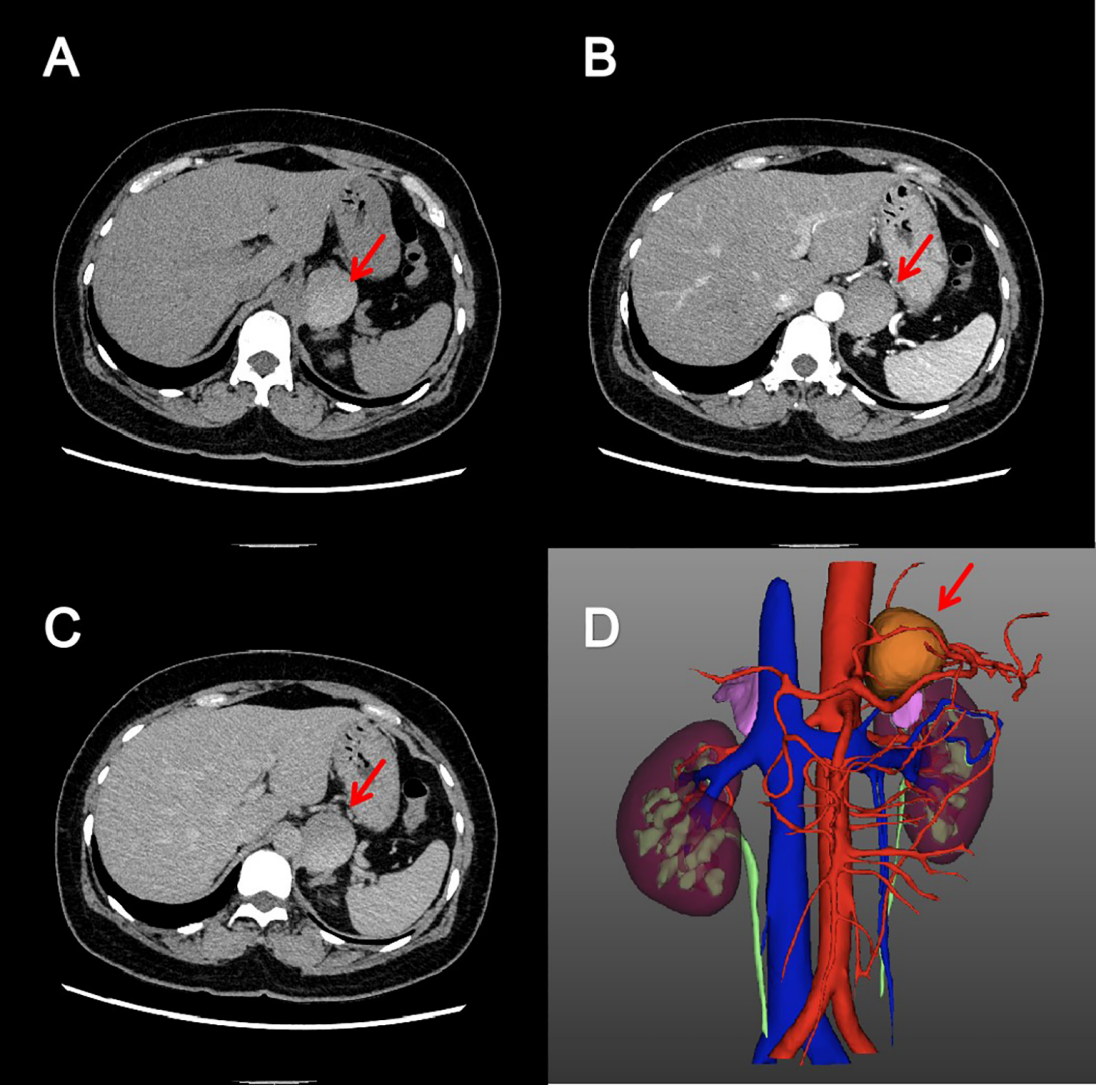
Adrenal enhanced computed tomography image. Axial pre-contrast **(A)**, arterial phase **(B)** and excretory phase **(C)** CT images showed a cystic, well-circumscribed mass without enhancement in the left adrenal region. The reconstructed three-dimensional image **(D)** showed that the mass was closely related to the left adrenal gland.

Screening for adrenal hormones, including adrenocorticotropic hormone, cortisol, and renin-aldosterone-angiotensin II levels measured at 00:00, 08:00, and 16:00, were all within normal limits. Furthermore, other biochemical studies, including complete blood count, coagulation function, liver function, renal function, serum electrolyte concentrations, and blood glucose, revealed no significant abnormalities. Key laboratory test results are shown in **Table 1**. Contrast-enhanced CT scan revealed a well-circumscribed, slightly hyperdense mass in the left adrenal region, measuring approximately 43×37×36 mm (**Figure 1**). The lesion was intimately associated with the left adrenal gland, exhibited peripheral calcification, and had a CT value of approximately 74 Hounsfield units. No significant enhancement was observed. Based on imaging characteristics, the mass was considered most consistent with a retroperitoneal cystic lesion. However, an adrenal tumor or a simple adrenal cyst could not be entirely excluded.

**Table 1:** Laboratory test results.

	Patient values	Reference values	Units
ACTH at 00:00 am	16.7	0-20	pg/mL
ACTH at 08:00 am	23.8	6-40	pg/mL
ACTH at 16:00 pm	16.4	3-30	pg/mL
VMA in 24-hour urine	60.28	0-60.6	μmol/24h
Cortisol at 00:00 am	277.46	-	nmol/L
Cortisol at 08:00 am	358.56	-	nmol/L
Cortisol at 16:00 pm	180.23	-	nmol/L
Renin	19.81	3.80-38.8	pg/mL
Aldosterone	226.72	40-310	pg/mL
Angiotensin II	105.58	49-252	pg/mL
WBC	5.42	3.5-9.5	×109/L
Neutrophils	2.63	1.8-6.3	×109/L
Lymphocytes	2.27	1.1-3.2	×109/L
Monocytes	0.42	0.1-0.6	×109/L
Eosinophils	0.07	0.02-0.52	×109/L
Basophil	0.03	0-0.06	×109/L
Blood glucose	5.7	3.9-6.1	mmol/L

ACTH, Adrenocorticotropic hormone; VMA, Vanillylmandelic acid; WBC, White blood cell.

**Figure 2:**
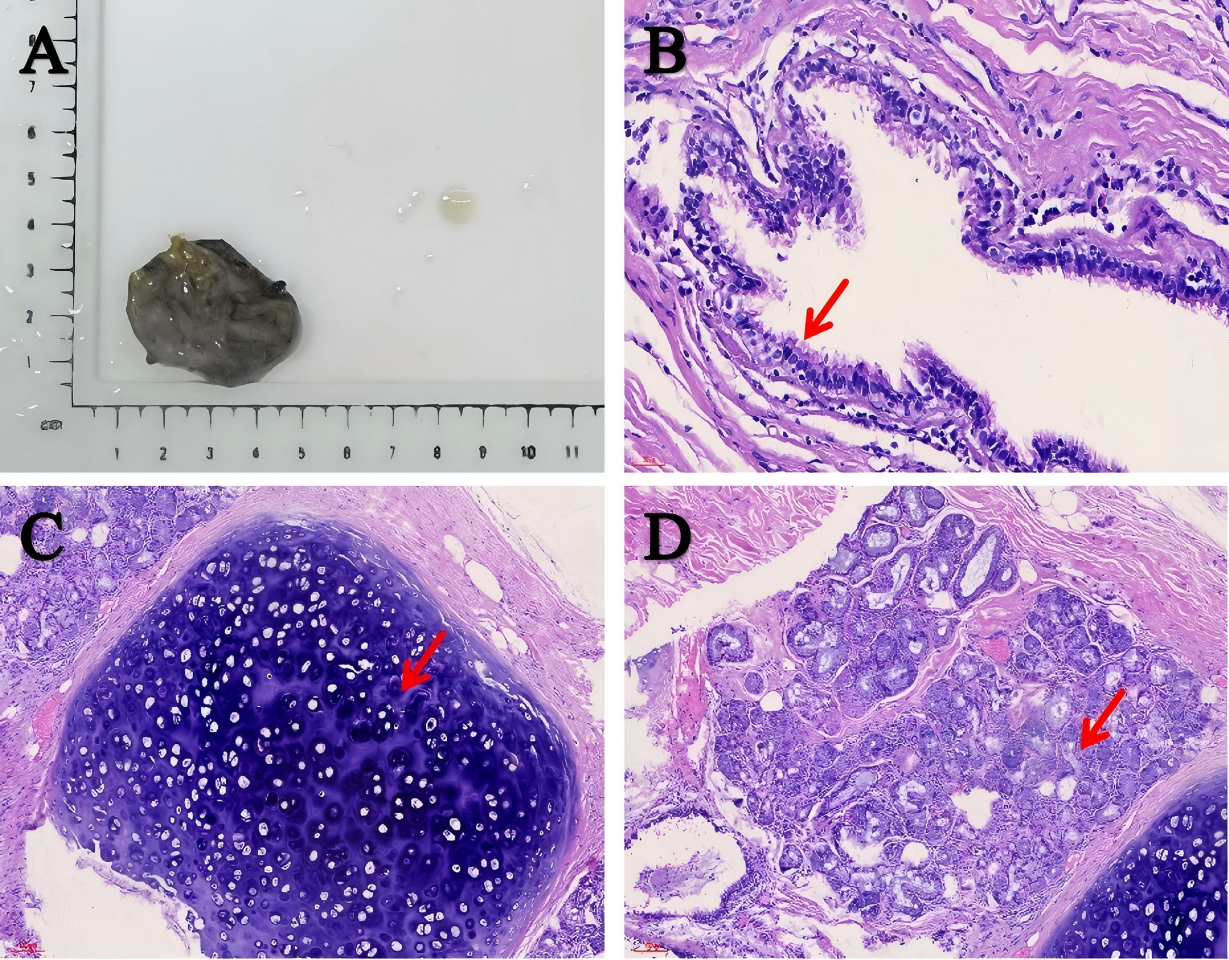
Macroscopic and microscopic manifestations of the cyst. **(A)** The cyst wall was dark gray and smooth. **(B–D)** Histopathological analysis was consistent with the diagnosis of bronchogenic cyst. The cyst wall was covered with ciliated columnar epithelial cells (scale bar: 30 μm). The arrow shows the structure of ciliated columnar epithelium **(B)**. The cyst wall was composed of fibrous connective tissue. Bronchial cartilage and mucous glands were found under the epithelium, accompanied by inflammatory cell infiltration (scale bar: 100 μm). The arrow shows the cartilage **(C)** and gland **(D)** structure.

For definitive diagnosis, the patient underwent robot-assisted laparoscopic resection of the left retroperitoneal lesion via a transabdominal approach. During the surgical exploration, no adhesions were found in the abdominal viscera, and adjacent organs such as the spleen and colon showed no abnormalities. A 3.5×2.5×0.5 cm cyst was found in the left retroperitoneal area adjacent to the upper pole of the left kidney and closely abutting the adrenal gland. The capsule was intact and the surface was smooth, consistent with the cystic manifestations suggested by preoperative imaging. Upon puncture, the cyst yielded a small amount of white, viscous fluid. Macroscopically, the cyst wall was dark gray with a smooth surface (**Figure 2A**) and a uniform thickness of 0.2 cm. Microscopic examination revealed that the cyst wall was lined by ciliated columnar epithelium and composed of fibrous connective tissue (**Figure 2B**). Bronchial cartilage (**Figure 2C**) and subepithelial mucinous glands (**Figure 2D**) were identified within the cyst wall, along with numerous infiltrating lymphocytes and plasma cells, confirming the diagnosis of a bronchogenic cyst. Postoperative abdominal CT confirmed complete excision. Three days postoperatively, the patient’s condition improved, and she was discharged without complications. At the 6-month follow-up, she remained asymptomatic with no evidence of recurrence.

## Discussion

**Figure 3:**
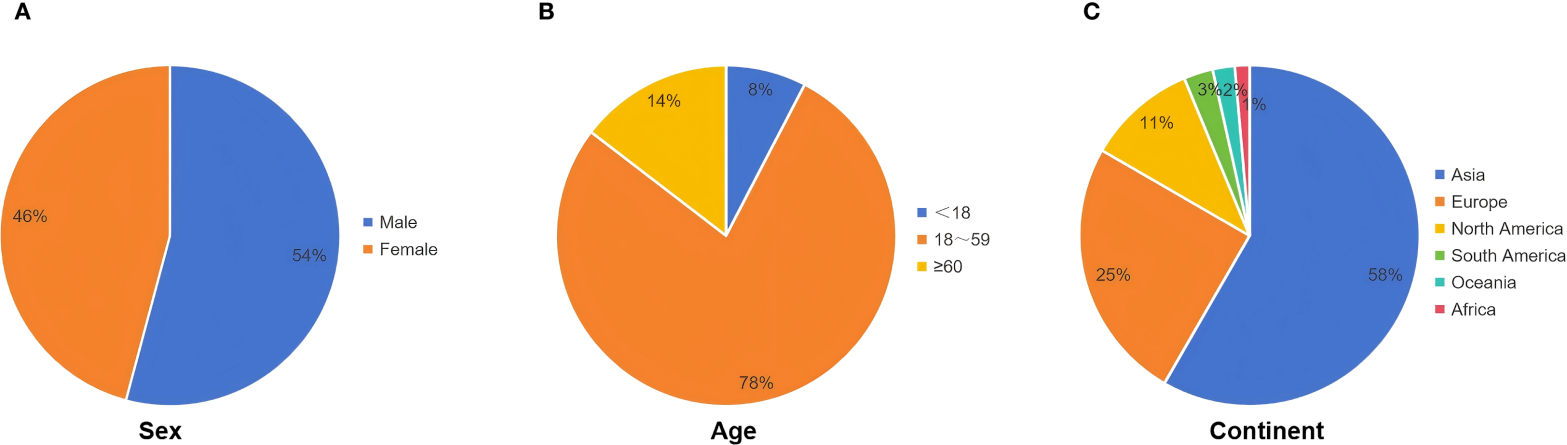
Baseline characteristics of patients in our literature review. **(A)** Gender composition of patients. **(B)** Age composition of patients (year). **(C)** Geographical distribution of patients.

Bronchogenic cysts are congenital developmental abnormalities originating from the primitive foregut and are classified as foregut cysts (6). The cyst wall typically consists of ciliated epithelium, cartilage, smooth muscle, and mucous glands. The cyst contents are usually serous or mucoid but may become purulent if infected (3, 7). During the 3rd to 7th week of embryonic development, abnormalities in the development of the primitive foregut into the trachea and lung buds can occur. This process can lead to detachment of respiratory epithelial cells, which may proliferate aberrantly in ectopic sites, ultimately forming cysts (1, 8). This developmental process may be related to genetic or environmental factors, but the exact pathogenesis remains unclear (9). Based on anatomical location, bronchogenic cysts are classified into mediastinal, intrapulmonary, and ectopic types (8). Mediastinal and intrapulmonary cysts are more common, often occurring in mediastinal regions adjacent to the trachea or esophagus, or within the pulmonary parenchyma (10). Ectopic cysts, which are rarer, may occur in extrathoracic locations, such as the spinal canal and abdominal cavity, potentially leading to misdiagnosis as other cystic lesions (11–13). The exact prevalence is unknown. Although most cases are identified in children and adolescents, diagnosis in adults is not uncommon (14, 15). Generally, there is no significant gender predilection, although some reports indicate a slightly higher incidence in males (16). Retroperitoneal bronchogenic cysts are even rarer, first reported by Miller et al. in 1953, and account for only 0.03% of all cases (2, 17). They are thought to arise when an embryonic bronchial tree malformation results in a bronchial bud being severed by the developing diaphragm, subsequently migrating into the abdominal cavity and forming a retroperitoneal bronchogenic cyst (1, 8). To systematically review this rare entity, we searched PubMed, Embase, and Scopus databases using the keywords “retroperitoneal,” “bronchogenic,” and “cyst.” After screening titles and abstracts, we identified 126 articles published between 2000 and 2024, encompassing the clinical characteristics of 144 patients with retroperitoneal bronchogenic cysts. Key clinical characteristics can be found in **Table 2**. Among the 144 patients, 54% were male and 46% were female, consistent with previous reports of a slight male predominance (**Figure 3A**). The age at diagnosis ranged from prenatal to 78 years, with a mean age of 42.4 years. The majority (92%) were diagnosed in adulthood (**Figure 3B**). Most reported patients were from Asia and Europe, accounting for 58% and 25%, respectively (**Figure 3C**).

**Table 2:** A literature review of retroperitoneal bronchogenic cyst.

	First author	Country	Year	Sex	Age	Location	Size (cm)	Chief complaint	Therapy	Ultrasound features	CT features	MRI features	Macroscopic features	Content	Epithelium	Cartilage	Glands	Muscle	Follow-up (months)	Outcome
1	O Reichelt	Germany	2000 (35)	M	46	Right adrenal gland	3.8×3.5	Asymptomatic	Surgical resection	Solid-appearing mass	NA	Cystic mass	Smooth outer surface	Yellow mucinous fluid	Ciliated pseudostratified columnar	✓	Seromucous	✓	NA	NA
2	P Bagolan	Italy	2000 (36)	M	3 m	Right adrenal gland	3.5×2.5×4.5	Asymptomatic	Surgical resection	Anechoic cystic lesion with a regular outline and clearly defined margins	Low-density (fluid-filled) lesion	NA	NA	NA	Ciliated pseudostratified	✓	Seromucous	✓	NA	NA
3	R Murakami	Japan	2000 (29)	M	27	Left adrenal gland	6	Asymptomatic	Laparotomy	Hypoechoic mass	Nonenhancing cystic mass with smooth, sharp margins that were clearly demarcated from the surrounding structures	Low and intermediate signal intensity on T1, high signal intensity with a rim of low signal intensity on T2	Oval	Thick brown mucoid material	Ciliated pseudostratified columnar	NA	Seromucous	NA	NA	NA
4	M Montesino	Spain	2000 (37)	M	38	Left adrenal gland	NA	Left lumbar pain	Surgical resection	NA	NA	NA	NA	NA	NA	NA	NA	NA	NA	NA
5	W J Haddadin	UK	2001 (38)	M	51	Left adrenal gland	5	Persistent epigastric pain	Surgical resection	NA	Well defined and circumscribed solid mass	NA	Cystic mass	Thick whitish mucoid fluid	Ciliated pseudostratified columnar	✓	Seromucous	NA	NA	NA
6	Mark I	USA	2001 (39)	M	33	Left adrenal gland	6×5	Left flank pain and gross hematuria	Surgical resection	Mass	Mass with an attenuation of 62 HU	Homogeneously isointense illumination on T1 and brightly hyperintense illumination on T2	Fluctuant mass with a smooth exterior surface	NA	Ciliated	✓	Bronchial	✓	NA	NA
7	Ryuichiro O	Japan	2001 (40)	F	67	Left subdiaphragmatic	16×12	Abdominal fullness	Surgical resection	Cyst contained not only fluid but also floating contents	Cystic mass whose wall contained solid portions and was slightly enhanced	NA	Encapsulated cystic tumor	Reddish gelatinous contents	Ciliated columnar	NA	Seromucous	✓	26	No recurrence
8	Akira I	Japan	2002 (41)	F	46	Left subdiaphragmatic	4	Left-arm numbness	Surgical resection	NA	Nonenhancing cystic mass	Lower signal intensity than fat on T1 and much higher than fat on T2	NA	Mucoid material	Respiratory	✓	Mucous	✓	NA	NA
9	David J	Australia	2002 (42)	F	8	Left adrenal gland	5×2.4×2	Central colicky abdominal pain	Laparoscopic	Quadrilateral-shaped mass with a well-defined capsule	Rhomboid mass with uniform internal structure with minimal enhancement	NA	NA	NA	Respiratory	✓	Seromucous	✓	24	No recurrence
				M	15	Left adrenal gland	5×4.8×3	Lower left posterior thoracic pain	Laparoscopic	NA	Nonenhancing, calcified and well-defined mass	NA	NA	NA	Respiratory	✓	Seromucous	✓	48	No recurrence
10	R Martín	Spain	2002 (43)	M	51	Left subdiaphragmatic	8×5	Asymptomatic	Surgical resection	Cyst	Cystic mass with smooth contours and clear demarcation from neighboring tissues (60 HU)	Well-circumscribed cystic mass with high signal	Nodular, well encapsulated and cystic mass	Dark red-brown hemorrhagic and seromucinous fluid	Ciliated pseudostratified	✓	Bronchial	✓	NA	NA
11	Takahara K	Japan	2002 (44)	M	65	Retroperitoneal	11	Abdominal pain	Surgical resection	Mass	Mass	Mass	NA	NA	Ciliated pseudostratified columnar	NA	NA	✓	NA	NA
12	Norio T	Japan	2002 (45)	M	50	Posterolateral left kidney	3.5×2.5	Asymptomatic	Surgical resection	Internal low echo and almost uniform cystic mass	Internal homogeneous mass with slightly higher CT value than liver	Almost equal signal to skeletal muscle on T1 and high signal on T2	Round cyst with smooth surface	NA	Ciliated columnar	✓	Bronchial	NA	NA	NA
13	N Hamaguchi	Japan	2002 (46)	M	39	Left subdiaphragmatic	5×2.5×2.2	Asymptomatic	Surgical resection	NA	NA	NA	Cyst	White turbid mucus	Ciliated	✓	Mucous	✓	NA	NA
14	E Hisatomi	Japan	2003 (47)	M	42	Left subdiaphragmatic	12×10×10	Left flank pain	Surgical resection	NA	Well-circumscribed, nonenhancing and multilocular cystic mass of water density	Multiloculated mass with a low signal intensity on T1 and very high signal intensity on T2	Oval cystic structure	Thick, whitish-yellow, mucoid fluid	Ciliated pseudostratified columnar	✓	Seromucous	NA	NA	NA
15	Y C Kim	Korea	2003 (48)	M	45	Posterior surface of the left lobe of the liver	6	Asymptomatic	Surgical exploration	Homogeneous, echogenic mass	Homogenous mass	Mass	Cystic mass	Yellow viscid material	Ciliated pseudostratified columnar	✓	NA	✓	NA	NA
16	Jun M	Japan	2003 (49)	M	62	Posterior wall of the stomach	10×3×3	Asymptomatic	Surgical resection	Multicystic mass	Multicystic lesion	NA	Soft, fusiform mass	Brownish serous fluid or clear mucus	Ciliated pseudostratified columnar	✓	Mucous	✓	NA	NA
17	Nasim H	USA	2003 (50)	F	59	Left adrenal gland	7×5	Asymptomatic	Laparoscopic	Uniformly echogenic mass	Homogeneous mass	NA	Cystic mass	Thick mucin	Ciliated pseudostratified	✓	Mucous	✓	NA	NA
18	Roland A	Sweden	2003 (51)	M	38	Pancreas	3.5	Upper abdominal pain	Surgical resection	Cystic lesion	Cystic lesion	NA	Well delineated cyst	Mucinous content	Ciliated pseudostratified columnar	✓	Mucinous	✓	24	No recurrence
19	Tomomoto I	Japan	2003 (32)	F	41	Left adrenal gland	5.5×5.2×9.2	Left flank pain	Laparoscopic	Cystic mass	Homogeneously dense cystic mass	Well circumscribed mass with high signal intensity on T1 and T2	NA	NA	Ciliated pseudostratified columnar	✓	Seromucous	✓	NA	NA
20	Chatti, K	Tunisie	2003 (52)	M	51	Left adrenal gland	4	Abdominal pain	Laparoscopic	Cystic	Mass without enhancement	NA	NA	NA	Ciliated columnar	✓	✓	NA	NA	NA
21	Brian K	Singapore	2004 (53)	F	29	Common hepatic and common bile ducts	13×8×18.9	Asymptomatic	Laparotomy	NA	Cyst	NA	NA	NA	Ciliated pseudostratified columnar	✓	NA	✓	NA	NA
22	Hashine K	Japan	2004 (54)	M	30	Left adrenal gland	8	Asymptomatic	Laparoscopic	NA	Cyst	Cyst	NA	NA	Respiratory	✓	Mucous	✓	NA	NA
23	Hidetoshi E	Japan	2004 (55)	M	59	Dorsal side of pancreatic body	8×7.5×7	Back pain	Surgical resection	NA	Well-defined, circumscribed multilobular mass	Mass lesion with low signal intensity on T1, high signal intensity on T2	Spherical cystic mass	Viscous, white or dark brown fluid	Ciliated pseudostratified columnar	NA	NA	✓	12	No recurrence
24	O Ishizuka	Japan	2004 (56)	M	36	Left adrenal gland	5×3	Asymptomatic	Laparoscopic	NA	Soft tissue mass	Mass with signal intensity slightly higher than liver on T1 and much higher than liver on T2	NA	NA	Ciliated pseudostratified columnar	NA	NA	NA	NA	NA
25	Hideaki K	Japan	2005 (57)	M	59	Left adrenal gland	7×3.5×3	Asymptomatic	Surgical resection	Cystic tumor	Dumbbell-shaped cyst with dense fluid	Dumbbell-shaped cyst with dense fluid	Gourd-shaped cyst with a smooth surface	Amber, turbid and high-viscosity fluid	Ciliated columnar	NA	Seromucous	✓	NA	NA
26	Mike K	USA	2005 (3)	F	46	Left paraspinal region	4×3.5×3.5	Left flank pain	Surgical resection	NA	Mass with a thin enhancing wall	NA	Soft spherical mass	Tan-yellow, homogenous gelatin	Ciliated columnar	✓	NA	NA	NA	NA
27	Seung S	Korea	2005 (58)	M	59	Peripancreatic	7	Asymptomatic	Surgical resection	NA	Unilocular cystic mass	NA	Cystic mass with smooth and glistening outer surface	Thick brownish mucoid material	Ciliated pseudostratified columnar	NA	NA	NA	NA	NA
28	Sang Y	Korea	2005 (59)	F	62	Gastroesophageal junction	1.7	Asymptomatic	Surgical resection	Oval echogenic mass	Homogeneous, low-density nodule	NA	NA	Brown, soft, gelatinous tissue	Ciliated pseudostratified columnar	NA	Seromucinus	✓	NA	NA
29	H Frickmann	Germany	2006 (60)	M	38	Left adrenal gland	5×3.5×2.5	Asymptomatic	Surgical resection	NA	NA	NA	NA	Yellow soft content	Ciliated pseudostratified columnar	NA	Mucous	✓	NA	NA
30	Shin-E W	China	2006 (25)	M	69	Above the pancreas body	7×3.5×2	Right upper quadrant discomfort	Laparotomy	Cystic mass	Nonenhancing cystic mass	NA	NA	Mucin content	Ciliated	✓	Bronchial	✓	NA	NA
				F	54	Above the pancreas body	3×3×1	Asymptomatic	Surgical resection	Cystic mass with internal echogenic septum	NA	Thin-walled cystic mass with low signal intensity on T1, hyperintensity compared to cerebral spinal fluid on T2	NA	Viscous yellow fluid	Ciliated columnar	NA	NA	✓	NA	NA
31	Won-Min J	Korea	2006 (61)	M	33	Left adrenal gland	3×2×2	Asymptomatic	Surgical resection	NA	NA	Low density mass-like lesion	Cystic mass	Gelatinous fluid	Ciliated pseudostratified columnar	✓	NA	✓	NA	NA
32	Molnár B	Hungary	2006 (62)	F	18	Left adrenal gland	6×8	Thinness	Laparoscopic	NA	NA	NA	Cyst	Light grey, mucinous liquid	Ciliated pseudostratified columnar	NA	Mucous	✓	NA	NA
33	E.Y Kim	Korea	2007 (28)	M	17	Dorsal aspect of the pancreatic tail	3.2	Abdominal pain	Surgical resection	NA	Round, hypoattenuating cyst	Cyst with a thin wall	Cystic lesion with a smooth outer surface	Serous fluid and creamy material	Ciliated pseudostratified	NA	Mucous	✓	NA	NA
34	Franco O	Chile	2007 (63)	F	24	Adhered to the pancreas	9×4.5×3.5	Abdominal pain	Laparotomy	Cystic lesion	Cystic mass with clear boundary	NA	Cyst	Chocolate-like mucus	Ciliated pseudostratified columnar	✓	NA	✓	NA	NA
35	Majda V	Croatia	2007 (64)	M	53	Peripancreatic	8×6×6	Epigastric pain	Surgical resection	Cystic lesion	Well-defined and circumscribed cystic mass with dense fluid content	NA	Smooth outer surface and glistening	NA	Ciliated pseudostratified columnar	NA	Seromucous	✓	6	No recurrence
36	Elizabeth T	Georgia	2007 (65)	F	75	Left adrenal gland	5	Abdominal pain	Laparoscopic	NA	Mass (100 HU)	NA	Fleshy, soft, cystic mass	NA	Ciliated columnar	NA	NA	✓	NA	NA
37	Pei-Yi C	China	2007 (66)	M	55	Left adrenal gland	4×3	Asymptomatic	Laparoscopic	NA	Solid tumor	NA	Cystic mass	Thick proteinaceous fluid	Ciliated pseudostratified columnar	✓	NA	NA	NA	NA
38	Sadatsugu M	Japan	2007 (67)	M	39	Left adrenal gland	3.5×3	Low-grade fever	Laparoscopic	Homogenous solid mass	Homogenous solid mass	NA	NA	Brown and slightly turbid liquid	Pseudostratified columnar	✓	Mucous	NA	NA	NA
39	Y Zoubeir	Maroc	2007 (68)	F	57	Left renal	5×3	Hypodynamia	Surgical resection	Low echo mass	Cystic lesion	Homogeneous lesion	Cyst	Mucus-like substance	Respiratory	✓	Mucous	✓	6	No recurrence
40	Huang M	China	2008 (69)	M	54	Left suprarenal	NA	Asymptomatic	Laparoscopic	NA	Cystic	NA	Cystic mass	NA	Ciliated pseudostratified columnar	NA	NA	✓	NA	NA
41	Andres R	USA	2008 (70)	M	40	Left adrenal gland	6.2	Asymptomatic	Laparoscopic	NA	NA	NA	NA	NA	NA	NA	NA	NA	24	No recurrence
42	F F Onol	Turkey	2009 (71)	M	36	Left adrenal gland	6×5.8×5	Asymptomatic	Surgical resection	Mass	Lobulated mass with heterogeneous intensity and calcification	Lobulated mass with heterogeneous intensity and calcification	Rubbery cystic mass with a smooth wall	Mucoid, brownish fluid	Ciliated pseudostratified columnar	NA	Seromucous	✓	NA	NA
43	Jae M	Korea	2009 (72)	F	41	Left adrenal gland	4.8×3.5×4.2	Asymptomatic	Laparoscopic	NA	Ovoid-shaped homogeneous mass	NA	Slightly lobulated rubbery mass	Brownish granular material	Ciliated pseudostratified columnar	✓	Seromucous	✓	NA	NA
44	Jorge O	USA	2009 (73)	M	67	Left subdiaphragmatic	3.9×3.7	Asymptomatic	Laparoscopic	Hypoechoic mass with internal, diminutive, intensely hyperechoic foci	Mass	Mass	NA	NA	Ciliated columnar	✓	Mucous	✓	NA	NA
45	M Manz	Schweiz	2009 (7)	M	49	Left subdiaphragmatic	5	Asymptomatic	Surgical resection	Cystic mass	Homogeneous, well-defined mass	NA	NA	NA	Ciliated	✓	Bronchial	✓	NA	NA
46	A S Valero	Spain	2009 (74)	M	26	Left subdiaphragmatic	7.6×4.4	Asymptomatic	Laparotomy	NA	Lesion with clear boundary and low density	NA	NA	NA	Ciliated pseudostratified columnar	NA	NA	✓	NA	NA
47	Raphael E	USA	2010 (75)	M	44	Left adrenal gland	3.1×1.6	Asymptomatic	Laparoscopic	NA	Homogeneous mass (42 HU)	Smooth, bilobed mass of high signal intensity on T1 and T2	NA	NA	Ciliated columnar	NA	✓	NA	NA	NA
				F	32	Neck of the pancreas	2.6	Abdominal pain	Surgical resection	Cystic, hypoechoic mass	Lobulated, well-circumscribed homogeneous low-attenuation mass	Lesion with increased signal on T2 and intermediate-to-decreased signal on T1 and no appreciable enhancement	NA	NA	Ciliated	NA	Mucinous	✓	NA	NA
48	Ho G	Korea	2010 (76)	F	20	Stomach	2.5×2	Asymptomatic	Laparoscopic	NA	Mass	NA	NA	NA	Pseudostratified	NA	NA	✓	NA	NA
49	Kazuki I	Japan	2010 (77)	F	64	Lesser curvature of the stomach	3×4×2	Asymptomatic	Laparoscopic	Cystic mass	Well-circumscribed cystic mass	NA	Cystic mass with smooth surface	Mucous fluid	Ciliated pseudostratified	NA	NA	✓	NA	NA
50	O Rud	Germany	2010 (78)	F	51	Left adrenal gland	3.5×2.5×2.5	Asymptomatic	Laparoscopic	NA	Well-circumscribed cystic mass (70–80 HU)	Oval mass with high signal on T1 and T2	Partial cystic tumor	NA	Ciliated columnar	✓	Mucous	✓	9	No recurrence
51	Rafael D	Spain	2010 (79)	M	67	Gastroesophageal junction	6	Low back pain	Laparoscopic	NA	Cystic oval mass	NA	NA	NA	Ciliated	NA	NA	NA	NA	NA
52	Sugar I	Hungary	2010 (80)	M	49	Left adrenal gland	6×3×4	Asymptomatic	Laparoscopic	NA	Soft tissue mass (35 HU)	NA	NA	Thick gel-like substance	Ciliated columnar	✓	NA	✓	NA	NA
53	Adolfo P	Italy	2010 (81)	M	33	Ileal mesentery	3.2	Abdominal pain	Laparotomy	Mass	NA	NA	Mass	NA	Ciliated pseudostratified columnar	NA	Mucinous	✓	NA	NA
54	Yun-Mee C	Korea	2010 (82)	M	12	Right subdiaphragmatic	14	Periumbilical pain	Laparotomy	Mass	Mass	NA	Cystic mass	Yellowish mucoid material	Ciliated pseudostratified columnar	NA	NA	NA	9	No recurrence
55	Zdichavsky M	Germany	2011 (83)	F	27	Right adrenal gland	4.5×9×5	Asymptomatic	Laparoscopic	Mass	Mass	NA	Cystic	NA	Respiratory	NA	NA	✓	NA	NA
56	Ziyad A	USA	2012 (84)	F	23	Right adrenal gland	4	Asymptomatic	Robotic	NA	Bilobed mass (24 HU)	Mass	NA	NA	NA	NA	NA	NA	NA	NA
57	C Brient	France	2012 (33)	M	60	Left adrenal gland	NA	Asymptomatic	Expectant treatment	NA	Mass (60 HU)	Hypersignal intensity on T1 and intermediate signal intensity on T2	NA	NA	Ciliated pseudostratified columnar	NA	Seromucous	NA	36	No change
58	Fazl Q	India	2012 (85)	F	30	Right lobe of liver	10×8×8	Abdominal pain	Laparotomy	Cystic lesion	Unilocular cyst	NA	NA	Mutinous fluid	NA	NA	NA	NA	NA	NA
59	Kim G	Belgium	2012 (86)	M	48	Left adrenal gland	7×7.5×4.3	Thoracic discomfort	Surgical resection	Multilocular mass	Multilocular mass	Multilocular mass	Rubbery lesion	Mucoid or gelatinous material	Ciliated columnar	✓	Seromucous	NA	NA	NA
60	George P	UK	2012 (87)	M	50	Left adrenal gland	2.3	Asymptomatic	Laparoscopic	NA	Cystic lesion	NA	NA	NA	Respiratory	✓	Seromucinus	✓	NA	NA
61	Nicolas P	France	2012 (88)	M	77	Left adrenal gland	10×8.5×5	Asymptomatic	Surgical resection	NA	Bilobular cystic mass	High intensity on both TI and T2	NA	Brown liquid	Ciliated columnar	✓	NA	NA	NA	NA
62	Patrick B	USA	2012 (89)	F	23	Left adrenal gland	5.2×4	Abdominal discomfort	Laparoscopic	NA	Heterogeneous mass	NA	NA	Mucinous material	Ciliated	✓	NA	NA	NA	NA
63	Kang K	Korea	2012 (90)	M	61	Esophagogastric junction	3.5×2	Asymptomatic	Surgical resection	NA	NA	NA	NA	NA	NA	NA	NA	NA	NA	NA
				F	62	Esophagogastric junction	3.5×2.5×1.5	Abdominal pain	Surgical resection	NA	NA	NA	NA	NA	NA	NA	NA	NA	NA	NA
				F	45	Left adrenal gland	8	Abdominal discomfort	Surgical resection	NA	NA	NA	NA	NA	NA	NA	NA	NA	48	No recurrence
				F	45	Between the tail of the pancreas and the stomach	3.6	Asymptomatic	Surgical resection	NA	Thin-walled and homogeneous mass	NA	NA	Turbid white fluid	Ciliated pseudostratified	NA	NA	NA	NA	NA
64	Olof J	Germany	2013 (91)	M	50	Left adrenal gland	4	Left flank pain	Laparoscopic	NA	Mass	NA	Cystic lesion	NA	Ciliated pseudostratified	✓	Seromucous	✓	NA	NA
65	Kyosuke M	Japan	2013 (92)	M	66	Left adrenal gland	5.5	Asymptomatic	Surgical resection	NA	Cystic mass	Low intensity mass on T1	Cystic tumor with smooth surface	Yellow mucous liquid	Ciliated pseudostratified	✓	Bronchial	NA	12	No recurrence
66	Michael D	USA	2013 (93)	F	49	Head of the pancreas	12×9×7	Abdominal pain	Surgical resection	Mass with thickened cyst walls	Mass with both solid and cystic components	NA	Well encapsulated mass	Dense mucous	Ciliated pseudostratified	✓	Mucous	NA	NA	NA
67	Mitsuru K	Japan	2013 (94)	F	51	Left adrenal gland	4.5×3.5×3	Back pain	Laparoscopic	Mass	Mass	Mass with iso signal intensity with skeletal muscle on T1 and high signal intensity on T2	NA	Turbid and high viscocity liquids	Ciliated columnar	NA	NA	NA	NA	NA
68	R Castro	Portugal	2013 (95)	F	36	Pancreatic tail	8	Abdominal pain	Laparoscopic	Cystic lesion	NA	Thin-walled cystic mass with high signal intensity on T1 and intermediate signal intensity on T2	Cystic lesion with a smooth outer surface	NA	Ciliated pseudostratified columnar	✓	Mucous	NA	NA	NA
69	Tina R	Switzerland	2013 (96)	F	42	Left adrenal gland	5×3.6×4	Abdominal pain	Laparoscopic	Polycystic structure with anechoic and hyperechoic portions	Dense cystic mass	NA	Cystic and encapsulated lesion with smooth and clear boundaries	NA	Ciliated	✓	Seromucous	NA	NA	NA
70	Yunnan C	China	2013 (97)	F	26	Left adrenal gland	4×3.5	Asymptomatic	Surgical resection	Mass	Ovoid-shaped homogeneous mass (47 HU)	High signal intensity on T1 and T2	Cystic and encapsulated mass with smooth and clear boundaries	Thick yellow mucoid fluid	Ciliated pseudostratified columnar	✓	Seromucous	✓	NA	NA
				F	50	Adjacent to the left kidney	3	Left flank pain	Laparoscopic	Mass	Well-circumscribed and homogeneous mass (10 HU)	NA	Cystic mass	Mucous fluid	Ciliated pseudostratified columnar	✓	Seromucous	✓	NA	NA
71	Ali M	Iran	2014 (20)	M	23	Between spleen and the left kidney	20×20×20	Abdominal pain	Surgical resection	NA	Sharply marginated mass with septa and calcification	NA	Creamy ovoid cystic lesion with irregular surface	Thick, brownish secretions	Pseudostratified columnar	✓	Seromucous	✓	48	Asymptomatic
72	Biao D	China	2014 (98)	F	30	Left adrenal gland	1.5×2×2	Persistent fever	Laparoscopic	NA	Homogeneous, round, low-density cystic mass with slight enhancement	NA	Cystic structure with a complete capsule	White seromucinous fluid	Ciliated pseudostratified columnar	NA	Seromucous	NA	NA	NA
73	De-Hong C	China	2014 (19)	M	51	Bilateral adrenal gland	L 1.5×0.9 R 1.7×2.1	Intermittent vague headache	Laparoscopic	Bilateral suprarenal mass	Soft tissue density nodular shadows	NA	NA	NA	Ciliated columnar	NA	Seromucous	✓	NA	NA
74	Maly T	Czech Republic	2014 (34)	F	6	Pancreatic corpus and tail	15×5×5	Asymptomatic	Laparotomy	NA	NA	Cystic lesion	NA	NA	Ciliated columnar	✓	Mixed	NA	48	No recurrence
75	Zdichavsky M	Germany	2014 (99)	F	19	Para-rectal	4.5×9×4.5	Abdominal pain	Laparoscopic	NA	NA	Mass	Cyst	Yellowish brown secretion	Ciliated	NA	Mucous	✓	NA	NA
76	Tomohiro T	Japan	2014 (100)	M	27	Left adrenal gland	5	Asymptomatic	Laparoscopic	Cystic pattern	Circumscribed mass (50 HU)	ISO-intensified tumor by T1 images and no T2-hyperintense signal	Cystic tumor	NA	Ciliated columnar	NA	Mucous	✓	NA	NA
77	Duygu H	Turkey	2015 (101)	M	42	Diaphragmatic crura	9×6×6	Back pain	Surgical resection	NA	Hypodense lesion (20 HU)	Multilocular cyst with hyperintense signal on T1	NA	NA	NA	NA	NA	NA	NA	NA
78	F P Robertson	UK	2015 (102)	M	56	Right hemidiaphragm	6.6	Right hypochondrial pain	Laparotomy	NA	Cyst	NA	NA	NA	NA	NA	NA	NA	NA	NA
79	Deying Z	China	2015 (103)	M	8	Left adrenal gland	3.6×3.4×3.1	Asymptomatic	Laparoscopic	Mass with low density echo	Ovoid-shaped homogeneous mass (10 HU) and slightly enhanced (17 HU)	NA	Cystic mass	Semitransparent egg-white-like liquid	Ciliated columnar	NA	Seromucous	✓	21	No recurrence
80	Gulay B	Turkey	2015 (104)	F	25	Left adrenal gland	4	Left flank pain	Laparoscopic	Cystic mass	Well-demarcated, thin-walled, hypodense cystic mass	NA	Cystic lesion	Dark grayish mucoid fluid	Ciliated pseudostratified columnar	NA	Seromucous	✓	NA	NA
81	Huang H	China	2015 (105)	F	35	Left adrenal gland	5	Asymptomatic	Laparoscopic	Low to no echo	Inhomogeneous soft tissue density	NA	Quasi-circular cyst	Milky white viscous liquid	NA	NA	NA	NA	84	No recurrence
				F	33	Left adrenal gland	8×13	Asymptomatic	Laparotomy	Low to no echo	Inhomogeneous soft tissue density	NA	Oval cyst	Yellow-green viscous liquid	NA	NA	NA	NA	NA	NA
				F	31	Between the posterosuperior pancreas and the liver	5.5	Abdominal pain	Laparotomy	No echo	Homogeneous low density	NA	Oval cyst	Milky white viscous liquid	NA	NA	NA	NA	180	No recurrence
				F	50	Left anterior of pancreas tail	7	Abdominal pain	Laparotomy	No echo	Homogeneous low density	NA	Oval cyst	Gray pink jelly-like liquid	NA	NA	NA	NA	120	No recurrence
				M	36	Left adrenal gland	4.6	Asymptomatic	Laparoscopic	Low echo	Homogeneous soft tissue density	NA	Quasi-circular cyst	Yellow-white viscous liquid	NA	NA	NA	NA	120	No recurrence
				F	45	Left adrenal gland	6.7	Left lumbar pain	Laparoscopic	Low echo	Homogeneous soft tissue density	NA	Long strip cyst	Yellow-white viscous liquid	NA	NA	NA	NA	2	No recurrence
82	Han X	China	2015 (30)	F	36	Adjacent to the left kidney and the body of the pancreas	17×14×7.5	Back pain	Laparotomy	Mass	Nonenhancing irregularly-shaped mass	Mass with heterogeneous signals	NA	Yellowish-brown oil-like fluid	Ciliated pseudostratified columnar	✓	NA	✓	16	No recurrence
83	Munish T	India	2015 (106)	F	34	Right hypochondrium	10×6	Heaviness in the right flank	Laparoscopic	Cystic lesion	Cystic lesion	NA	NA	Yellow colored serous fluid	Columnar	NA	Mucous	✓	2 w	Asymptomatic
84	Xue J	China	2015 (107)	M	52	Left subdiaphragmatic	2.5×2.5×0.5	Asymptomatic	Laparoscopic	NA	Well-defined soft tissue mass	NA	NA	Milky fluid	Ciliated	✓	Bronchial	NA	NA	NA
85	Ye R	Korea	2015 (108)	M	57	Left adrenal gland	5×4	Asymptomatic	Laparoscopic	NA	Well-defined, ovoid-shaped mass without enhancement	NA	NA	NA	Ciliated pseudostratified columnar	NA	NA	NA	NA	NA
86	Padnani A	USA	2016 (109)	F	73	Left retroperitoneal	NA	Chronic flank pain	Laparoscopic	NA	NA	NA	NA	NA	Respiratory	NA	NA	NA	NA	NA
87	Arnaud P	France	2016 (110)	M	36	Retrorectal	10×5	NA	Laparotomy	NA	NA	Cystic lesion	NA	Mucous	Ciliated pseudostratified	NA	NA	NA	36	No recurrence
88	Min W	China	2017 (22)	F	48	Left adrenal gland	8×6×5.5	Epigastric pain	Laparoscopic	Hypoechoic, partly calcified mass	Hypodense mass	NA	NA	Thick hematoma and secreted materials	Respiratory	✓	Bronchial	NA	NA	NA
89	Briseño R	Mexico	2018 (111)	F	53	Left adrenal gland	7.1×4.3×6.0	Left renal fossa pain	Laparoscopic	NA	Non-enhancing cyst (37 HU)	Non-enhancing cyst with high signal intensity on T1	NA	NA	Respiratory	✓	NA	✓	NA	NA
90	Joshua T	USA	2018 (31)	M	52	Left adrenal gland	3×3.1×2.2	Asymptomatic	Biopsy	NA	Cystic lesion	NA	NA	NA	Ciliated columnar	NA	NA	NA	NA	NA
91	Mahmut B	Turkey	2018 (112)	F	38	Left adrenal gland	6.3×2.5×5.5	Abdominal pain	Laparoscopic	NA	NA	Well-defined, ovoid, heterogeneous lesion with hyperintense components	NA	Mucous and fluid	Ciliated pseudostratified	✓	Mucous	NA	NA	NA
92	Mase T	Japan	2018 (113)	F	38	Left adrenal gland	8.6×8.1	Asymptomatic	Surgical resection	Cyst	Well-defined tumor	NA	NA	NA	NA	NA	Bronchial	NA	NA	NA
93	Shree V	India	2018 (114)	F	43	Left adrenal gland	8×7	Left hypochondial pain	Laparoscopic	NA	NA	NA	NA	NA	NA	NA	NA	NA	NA	NA
94	Qu L	China	2018 (115)	M	33	Left hepatic hilum	4.5	Asymptomatic	Robotic	NA	NA	NA	NA	NA	NA	NA	NA	NA	NA	NA
				M	78	Inferior of the left renal vein, left side of IVC	7	Asymptomatic	Robotic	NA	NA	NA	NA	NA	NA	NA	NA	NA	NA	NA
95	Alejandro J	Spain	2019 (116)	F	43	Lesser curvature of stomach	3×3.5	Abdominal pain	Laparotomy	Cystic lesion	Solid or cystic lesion with clear outline and homogeneous density	Cystic lesion	NA	Transparent liquid	Ciliated columnar	✓	Bronchial	✓	NA	NA
96	Cremona F	Italy	2019 (117)	M	32	Retropancreatic	NA	Asymptomatic	Laparoscopic	NA	NA	NA	NA	NA	NA	NA	NA	NA	NA	NA
97	Jayanthan B	India	2020 (118)	M	46	Left psoas muscle	4×3.4	Dyspeptic symptoms	Laparoscopic	NA	Solid spherical mass	NA	NA	NA	NA	NA	Seromucous	✓	NA	NA
98	A Yeon	Korea	2020 (119)	M	40	Presacral space	8×9.3×8.7	Asymptomatic	Laparoscopic	Unilocular cystic mass	Cystic mass with relatively homogeneous attenuation, less than 25 HU	NA	NA	Yellowish, viscous fluid	Ciliated pseudostratified columnar	NA	Seromucous	NA	NA	NA
99	Pietro A	France	2020 (120)	M	45	Pancreatic body and the left adrenal gland	9	Epigastric pain	Surgical resection	Cystic lesion	Well-defined round cystic lesion	Well-defined round cystic lesion	NA	Mucoid content	Ciliated	NA	NA	✓	NA	NA
100	Takazo T	Japan	2020 (121)	F	32	Left adrenal gland	3×1×4	Asymptomatic	Laparoscopic	NA	Multilocular cystic mass	Multilocular cystic mass	Cystic lesion	White mucus	Ciliated pseudostratified	✓	Mucous	NA	NA	NA
101	Vikash S	India	2020 (122)	M	30	Pre-aortic region in the epigastrium	5.9×4.2×4.2	Abdominal pain	Laparoscopic	Well-defined hypoechoic lesion with internal echoes	Well-defined thin-walled homogeneous hypodense cystic lesion	NA	Well-defined cystic lesion with both true and false capsules	White colored fluid	Respiratory	NA	NA	NA	NA	NA
102	Yang W	China	2020 (4)	M	27	Left adrenal gland	2.1×4.1	Asymptomatic	Laparoscopic	NA	Heterogeneous fusiform lesion with a clear boundary	NA	Viscous mass	NA	Ciliated pseudostratified columnar	✓	Seromucous	NA	NA	NA
				M	33	Right adrenal gland	3.1×5.9	Asymptomatic	Laparoscopic	Mixed echo mass	Fusiform heterogeneous lesion without enhancement	NA	NA	Grey mucus	Ciliated pseudostratified columnar	NA	NA	NA	NA	NA
103	P. Jayanthi	USA	2020 (123)	F	72	Pancreas	8.6×6×4.5	Recurrent hematochezia	Robotic	NA	Cystic mass	NA	Cyst	Brown to gray-green, inspissated, mucoid material	Ciliated pseudostratified	✓	NA	✓	NA	NA
104	Ji Q	China	2020 (124)	F	41	Left adrenal gland	3.9×3.2×3	Lumbar back discomfort	Laparoscopic	Cyst	Round cystic mass without enhancement	NA	NA	Myxoid material	Ciliated pseudostratified columnar	✓	NA	✓	NA	NA
105	Kaitao Y	China	2021 (5)	F	53	Left adrenal gland	3.3×2.7×3.5	Back pain	Laparoscopic	NA	Well circumscribed cystic lesion filling with non-enhancing fluid-density collections	Round small mass	NA	Mucinous content	Ciliated pseudostratified	✓	Seromucous	✓	24	No recurrence
106	Lei D	China	2021 (125)	F	17	Left adrenal gland	2.9×1.7×2.8	Epigastric pain	Laparoscopic	NA	Ovoid, well-defined, and homogeneous cystic lesion	Cystic mass of intermediate signal intensity on T1 and high signal intensity on T2	NA	Gray yellowish fluid	Ciliated pseudostratified columnar	✓	NA	NA	10	No recurrence
107	Elahe M	Iran	2021 (126)	M	20 d	Left adrenal gland	5×3.7×3.2	Asymptomatic	Surgical resection	Mass	Mass	NA	NA	NA	Respiratory	✓	NA	✓	9	No recurrence
108	Sarah C	New Zealand	2021 (127)	M	39	Left adrenal gland	3	Left colicky flank pain	Laparoscopic	NA	Adrenal incidentaloma (44 HU)	NA	NA	NA	Respiratory	✓	Seromucous	✓	NA	NA
109	Bi Y	China	2021 (9)	M	6	Left adrenal gland	4.5×2.8×8	Abdominal pain	Laparoscopic	NA	Well-defined, cystic, heterogeneous, no enhancement	NA	NA	Gelatinous substance	Ciliated pseudostratified columnar	✓	NA	✓	NA	NA
				M	18	Left adrenal gland	7.1×3.6×7	Asymptomatic	Laparoscopic	NA	Well-defined, soft tissue, heterogeneous, no enhancement	NA	NA	Mucoid substance	Ciliated pseudostratified columnar	✓	NA	✓	NA	NA
				M	27	Right adrenal gland	3.6×3.5×3.4	Asymptomatic	Laparoscopic	NA	Well-defined, soft tissue, heterogeneous, calcification, no enhancement	NA	NA	Mucoid substance	Ciliated pseudostratified columnar	✓	NA	✓	NA	NA
110	Han C	China	2022 (26)	F	57	Left adrenal gland	2.2×5.8	Asymptomatic	Laparoscopic	NA	Ovoid, well-defined, and heterogeneous lesion	NA	NA	Yellowish fluid	Pseudostratified columnar	✓	Seromucous	✓	12	No recurrence
111	Jessé C	Brazil	2022 (128)	F	66	Pancreatic body and the left adrenal gland	6.2×3	Asymptomatic	Surgical resection	Well-delimited solid-cystic lesion	Solid-cystic lesion with mild contrast enhancement	Heterogenous lesion	NA	NA	Ciliated pseudostratified columnar	✓	Seromucous	✓	NA	NA
112	Kenji A	Japan	2022 (129)	F	50	Left adrenal gland	7×5.1×3	Asymptomatic	Laparoscopic	NA	NA	Low-intensity tumor including a cystic lesion	NA	Gelatinized contents	NA	NA	Seromucous	✓	NA	NA
113	Daniel d	Brazil	2022 (24)	M	19	Retrorectal	5×4×4	Testicular pain	Surgical resection	NA	NA	Homogenous cyst	NA	NA	Ciliated pseudostratified columnar	NA	NA	NA	3 w	No recurrence
114	María d	Spain	2022 (130)	M	61	Pancreas	9.6×10.9×6.8	Asymptomatic	Surgical resection	Cystic mass	Cystic lesion	NA	NA	Green gel-like substance	Ciliated pseudostratified columnar	✓	Seromucous	✓	NA	NA
115	Norikazu T	Japan	2022 (131)	F	73	Left adrenal gland	2.8×1.4	Asymptomatic	Surgical resection	NA	High-density tumor	Hyperintense and hypointense/no signal drop and partial suppression/intermediate and intermediate signal intensity	NA	Mucous	Respiratory	NA	NA	NA	NA	NA
116	Takahiro A	Japan	2022 (132)	M	75	Left adrenal gland	3×1.5	Pollakiuria	Laparoscopic	NA	Nonenhancing mass with internal uniform high absorbance with marginal calcification	Mass with a strong signal on T1 and a weak signal on T2	Cystic lesion	Dark-brown liquid with a high coefficient of viscosity	Ciliated columnar	✓	Bronchial	✓	NA	NA
117	Takeshi T	Japan	2022 (133)	F	16	Left subdiaphragmatic	3.8	Abdominal pain	Laparoscopic	NA	Homogeneous cystic lesion	Cystic lesion with low signal on T1 and high signal on T2	NA	NA	Ciliated columnar	✓	Bronchial	NA	24	No recurrence
118	Yang Y	China	2022 (21)	F	49	Left adrenal gland	5.4×4	Asymptomatic	Laparoscopic	NA	Moderate density mass with a clear boundary (85 HU)	NA	Cystic	Brown jelly-like substance	Respiratory	✓	Mucous	✓	6	No recurrence
119	Bin Y	China	2023 (18)	F	24	Left adrenal gland	4.1×3.1×3.6	Asymptomatic	Laparoscopic	Cystic	Unilocular sacs, no calcification, no enhancement	NA	NA	Brown, thick secretions	Pseudostratified	NA	NA	NA	24	No recurrence
				M	33	Left adrenal gland	5.4×2.2×3.4	Asymptomatic	Laparoscopic	Hypoechoic solid	Multilocular sacs, massive calcification, no enhancement	NA	NA	Sediment-like calcifications	Pseudostratified columnar	NA	NA	NA	10	No recurrence
				F	26	Left adrenal gland	4.2×3.4×2.1	Asymptomatic	Laparoscopic	Hypoechoic solid	Multilocular sacs, punctate calcification, no enhancement	NA	NA	NA	Pseudostratified columnar	✓	NA	NA	11	No recurrence
120	Enze W	China	2023 (134)	F	52	Adjacent to aortaventralis	3.9×2.6	Asymptomatic	Laparoscopic	NA	NA	Oval cystic signal shadow with uniform signal and clear borders without enhancement (low signal on T1, high signal on T2)	NA	NA	Ciliated pseudostratified columnar	✓	Mucinous	NA	NA	NA
121	Ricardo E	Colombia	2023 (135)	F	41	Left adrenal gland	5.2×2.8	Asymptomatic	Laparoscopic	NA	Heterogeneous mass, with multiple calcifications	NA	NA	NA	Ciliated columnar	✓	NA	NA	NA	NA
122	Wei X	China	2023 (27)	M	48	Adjacent to the left renal artery	3×4×6	Back pain	Surgical resection	NA	Low-density cystic mass (30 HU)	Cyst with fluid-fluid level, no enhancement of cyst contents	NA	Viscous white liquid	Ciliated pseudostratified columnar	✓	Bronchial	NA	36	No recurrence
123	Tao Li	China	2023 (136)	F	34	Left retroperitoneal	4×4.9×5.1	Asymptomatic	Laparoscopic	Echoless cystic lesions, visible capsule, no obvious blood flow signal	Quasi-circular soft tissue shadow	Equal T1 long T2 signal, clear boundary, no obvious enhancement	Cystic	NA	Ciliated columnar	NA	Bronchial	✓	NA	NA
124	Abdul M	China	2024 (17)	M	49	Upper posterior region of the pancreas	8.8×7.2×2.6	Asymptomatic	Laparoscopic	NA	Space-occupying lesion with minimal enhancement (36 HU)	NA	Cystic-solid	White, viscous liquid	Ciliated pseudostratified columnar	✓	✓	✓	NA	NA
125	Hajra I	USA	2024 (137)	M	18	Left adrenal gland	9.6×6.9	Left back pain	Laparoscopic	NA	Non-enhancing, homogenous, thin walled, well circumscribed mass	NA	Soft, round, smooth mass	White creamy fluid	NA	NA	NA	✓	NA	NA
126	Karol G	Poland	2024 (138)	F	55	Left adrenal gland	1.9×1.3×3.2	Back pain	Laparoscopic	NA	NA	Smooth contoured mass	NA	Gelatinous	Respiratory	✓	Seromucous	NA	NA	No recurrence

NA, Not available; M, Male; F, Female; m, Months; w, Weeks; d, Days; L, Left; R, Right; HU, Hounsfield units.

**Figure 4:**
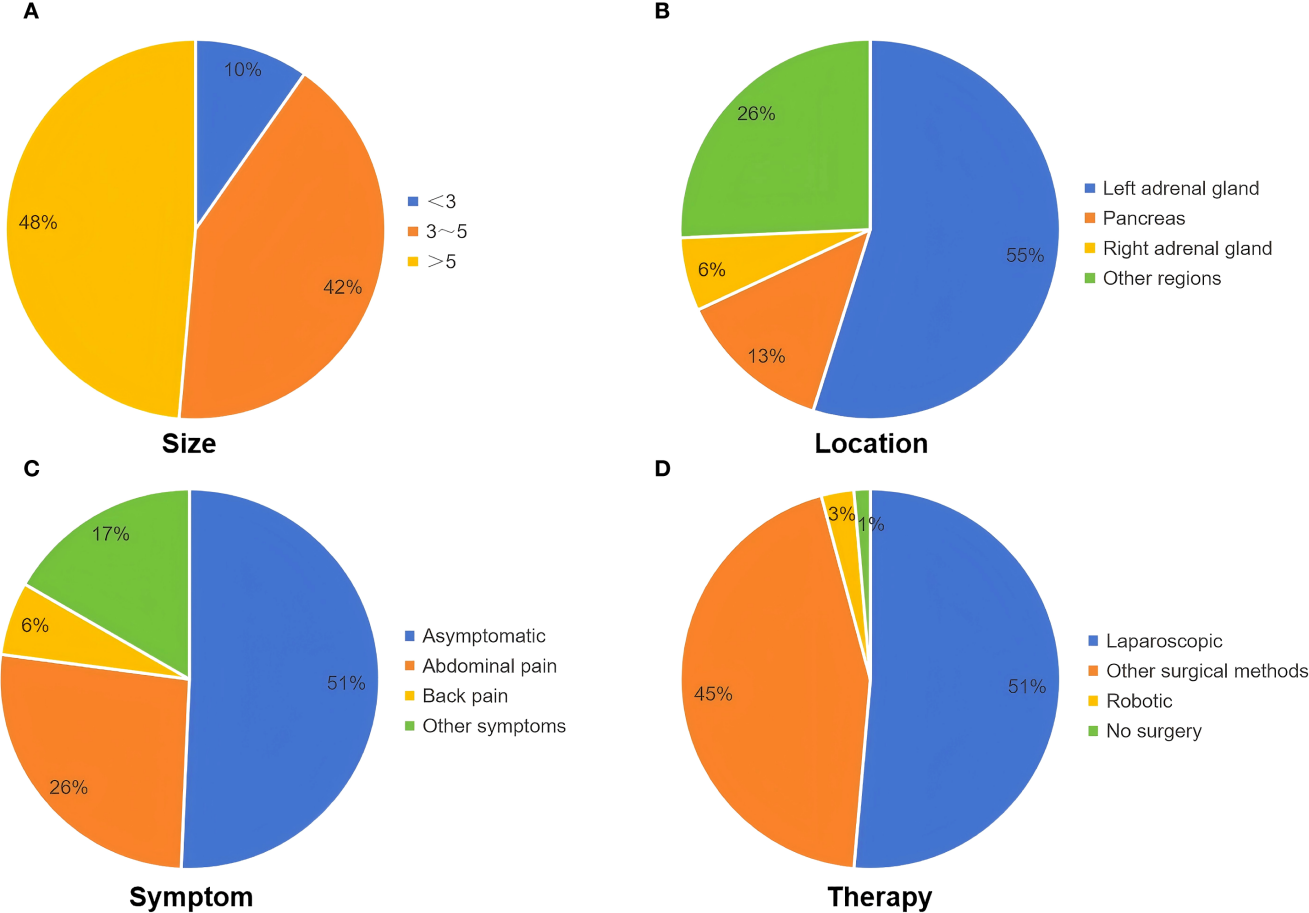
Clinical characteristics of patients in our literature review. **(A)** The range of the longest diameter of the cyst (cm). **(B)** The location of the cyst. **(C)** Symptoms of patients. **(D)** Treatment of patients.

Reports indicate that over 80% of cases occur in the left retroperitoneal region, particularly adjacent to the left adrenal gland, possibly due to the left pericardioperitoneal canal being larger and closing later than the right. The peripancreatic region is the second most common sites (5, 18). Our analysis showed that approximately 79% of retroperitoneal bronchogenic cysts were located on the left side, with about 55% of these found adjacent to the left adrenal gland. Cysts adjacent to the right adrenal gland accounted for approximately 6%, while those in the pancreatic area represented about 13% of reported cases (**Figure 4B**). Cao et al. reported an extremely rare case of a multilocular retroperitoneal bronchogenic cyst affecting both adrenal glands (19). To date, the mean diameter of reported retroperitoneal bronchogenic cysts is approximately 5.5 cm. The largest reported case, described by Mirsadeghi et al. in 2014, measured 20×20×20 cm (20). No significant correlation has been found between cyst size and patient age or gender. The distribution of the longest diameter of the cysts is shown in **Figure 4A**.

Most patients are asymptomatic, and the cysts are often discovered incidentally during routine imaging studies. However, when the cyst is sufficiently large to compress surrounding organs or results in complications such as infection, hemorrhage, or rupture, patients may present with symptoms such as abdominal or lumbar pain, fever, and gastrointestinal discomfort (e.g., nausea, vomiting) (4). Owing to nonspecific manifestations, diagnosis based solely on clinical features is difficult. Although these cysts are often adjacent to the adrenal glands or pancreas, they rarely cause abnormalities in adrenal hormones, electrolytes, or pancreatic enzymes (21). Few reported cases of retroperitoneal bronchogenic cysts have been associated with elevated levels of serum tumor markers, such as carbohydrate antigen (CA) 19–9 and CA 72-4 (22, 23). In a case reported by Bin et al., both serum CA 19–9 and CA 24–2 were elevated preoperatively and normalized following surgical resection (18). Although their clinical significance remains uncertain, tumor markers may assist in distinguishing benign from malignant retroperitoneal masses. In our review, approximately 51% were discovered incidentally and were asymptomatic. Approximately 26% of patients experienced abdominal pain, and approximately 6% presented with back pain, possibly due to mass effect (**Figure 4C**). A few patients reported nonspecific symptoms such as fatigue, weight loss, and gastrointestinal discomfort (e.g., dyspepsia and abdominal fullness). Two patients exhibited persistent fever, which resolved after surgery. Daniel et al. reported a case of a retrorectal bronchogenic cyst where the patient presented with testicular pain, which resolved postoperatively (24). Two patients presented with symptoms of pollakiuria and gross hematuria, although the association with the retroperitoneal bronchogenic cyst was unclear.

Given the nonspecific clinical presentation, most retroperitoneal bronchogenic cysts are discovered incidentally through imaging studies. However, no established imaging criteria currently exist for diagnosis, limiting the utility of imaging for preoperative confirmation. On ultrasound, retroperitoneal bronchogenic cysts typically appear as well-defined, anechoic lesions with no significant internal vascularity. However, the diagnostic value of ultrasound is often limited by interference from bowel gas (4, 17). The results of our literature review indicated that most retroperitoneal bronchogenic cysts appeared as hypoechoic or anechoic cystic masses on ultrasound, without specific manifestations. In contrast, CT and MRI are more effective and valuable modalities for preoperative assessment. On CT imaging, retroperitoneal bronchogenic cysts generally appear as round or oval homogeneous low-attenuation lesions, with CT values close to that of water. Calcification may be observed in the cyst wall in some cases (3, 25, 26). The cyst contents consist primarily of water and mucoproteins. However, variations in protein content, hemorrhage, or infection within the cyst can influence the CT attenuation values. Following intravenous contrast administration, the lesions typically demonstrate no enhancement (1, 27). On MRI, the cysts show low signal intensity on T1-weighted images (T1WI) and high signal intensity on T2-weighted images (T2WI). High protein content may cause high signal intensity on both T1WI and T2WI. Similar to CT findings, contrast-enhanced MRI typically shows no enhancement (21, 28). Additionally, fat-suppressed T1-weighted sequences help differentiate these cysts from malignant tumors such as teratomas and dermoid cysts. Both CT and MRI are capable of precisely determining lesion location and size. They also play a crucial role in evaluating the relationship between the cysts and adjacent organs, vessels, or nerves (29, 30). This information is essential for accurate cyst localization and surgical planning, facilitating effective treatment strategies. The findings of our literature review were also similar to the above-mentioned common imaging features on CT or MRI. Furthermore, endoscopic ultrasound (EUS) offers unique advantages for diagnosis. It provides clear visualization of the lesion’s location, morphology, internal structure, and relationship with adjacent tissues. This enables effective differentiation between retroperitoneal bronchogenic cysts and other retroperitoneal cystic lesions (e.g. pancreatic pseudocysts) or solid masses (e.g. stromal tumors). Importantly, EUS guidance allows for cyst aspiration or biopsy. This enables biochemical and cytological analysis, which is crucial for differentiating benign from malignant lesions (17, 22, 31). Retroperitoneal bronchogenic cysts must be differentiated from other retroperitoneal lesions, such as adrenal tumors, adrenal cysts, renal cysts, pancreatic pseudocysts, teratomas, neurogenic tumors, stromal tumors, lymphangiomas, and lymphomas (21). Ultimately, histopathological examination remains the gold standard for the definitive diagnosis of retroperitoneal bronchogenic cysts. The cyst wall is lined by respiratory epithelium, typically ciliated pseudostratified columnar epithelium. Additionally, the cyst wall stroma may contain cartilage, bronchial glands, and smooth muscle. Cysts lacking these elements are classified as foregut cysts (3). Notably, the case we reported exhibited characteristic pathological features consistent with a retroperitoneal bronchogenic cyst. Currently, complete surgical resection is the definitive treatment for retroperitoneal bronchogenic cysts. Early surgical intervention is recommended for patients with retroperitoneal bronchogenic cysts to relieve symptoms, establish a definitive diagnosis, and prevent complications such as hemorrhage, infection, or malignant transformation. Excision is also advised for asymptomatic patients (5, 32). In our review, the vast majority of patients with retroperitoneal bronchogenic cysts underwent surgical treatment; only two patients underwent biopsy with subsequent expectant management (31, 33). In recent years, with advancements in surgical techniques, laparoscopic surgery has proven safe and effective, associated with fewer complications and faster patient recovery. Approximately 51% of patients underwent laparoscopic surgery, and four patients underwent robot-assisted laparoscopic surgery (**Figure 4D**). Whether via traditional open surgery or the increasingly prevalent laparoscopic or robot-assisted laparoscopic approaches, complete excision of the lesion can be achieved. Most patients have favorable outcomes, confirming the safety and efficacy of surgical management (4, 21, 34). Additionally, to our knowledge, most patients have had a favorable prognosis, and there have been no reported cases of recurrence following complete surgical resection.

## Conclusion

In summary, retroperitoneal bronchogenic cysts are exceedingly rare. Their rarity and often non-specific clinical presentation pose significant challenges to early and accurate diagnosis. Histopathological examination remains the gold standard for definitive diagnosis. Surgical resection is the primary treatment modality, indicated for all patients regardless of clinical symptoms. Minimally invasive approaches (like laparoscopic surgery) are increasingly preferred due to their favorable safety and recovery profiles. Prognosis following surgical resection is generally excellent, with most patients achieving favorable long-term outcomes.

## Data Availability

The original contributions presented in the study are included in the article/supplementary material. Further inquiries can be directed to the corresponding authors.
